# Multifocal leukoencephalopathy in cocaine users: a report of two cases and review of the literature

**DOI:** 10.1186/s12883-015-0467-1

**Published:** 2015-10-19

**Authors:** Reza Vosoughi, Brian J. Schmidt

**Affiliations:** Section of Neurology, Department of Internal Medicine, College of Medicine, Faculty of Health Sciences, University of Manitoba, Winnipeg, Manitoba, R3A 1R9 Canada

**Keywords:** Cocaine, Leukoencephalopathy, Immune mechanism, Magnetic resonance imaging, Levamisole

## Abstract

**Background:**

Cocaine abuse is associated with several mechanisms of brain injury including ischemic, hemorrhagic and metabolic. Recently two case reports of leukoencephalopathy in cocaine users implicated a commonly used cocaine adulterant, levamisole. One well-documented adverse effect of levamisole, when used alone as antihelminthic or immunomodulatory drug, is multifocal inflammatory leukoencephalopathy. Therefore, immune mechanisms may also contribute to cocaine-induced brain injury.

**Case presentations:**

Two cocaine users with multifocal leukoencephalopathy, treated with steroids and plasmapheresis, are described. The first is a 25-year-old man who presented with unilateral motor and sensory impairment progressing to bilateral deficits, dysphagia, dysarthria and confusion over several days. Serial MRI showed increasing abnormal FLAIR signal lesions with patchy restricted diffusion and heterogenous enhancement deep in the right and left hemispheres, including periventricular white matter as well as in the pons and cerebellar peduncle. The second patient is a 41-year-old woman who presented with confusion and impaired balance. MRI showed bilateral periventricular FLAIR lesions with scattered restricted diffusion and subtle gadolinium enhancement of some of the lesions. She initially stabilized with supportive care only, but after further cocaine use was re-admitted six weeks later with marked neurological deterioration and MRI showed prominent worsening of the lesions. Both patients received steroid and plasma exchange and showed substantial improvement clinically and on imaging, which was sustained during out-patient follow-up.

**Conclusion:**

Multifocal leukoencephalopathy associated with cocaine use may have an inflammatory/immune basis, possibly related to levamisole contamination, at least in some patients. Three cases, including the present two, have been described wherein good neurological improvement was seen in association with steroid treatment. However, in the absence of appropriate clinical trials, it remains unknown whether immunotherapy is truly beneficial for these patients.

## Background

Three major categories of cocaine-induced brain injury have been described.

Vascular pathology is a well-recognized consequence of cocaine abuse and includes ischemic and hemorrhagic stroke. Underlying mechanisms include vasospasm, heightened sympathetic drive, acute hypertension, cardioembolism and, in rare cases, vasculitis [1]. Even in the absence of a history of cerebrovascular symptoms, compared to control populations an increased prevalence and severity of asymptomatic white matter lesions on magnetic resonance imaging (MRI) has been documented in cocaine users; these lesions are also thought to have an ischemic basis [2, 3].

A second mechanism of cocaine-induced brain injury is metabolic in nature. In particular, several cases of acute/subacute cocaine-induced leukoencephalopathy have been reported. Magnetic resonance imaging (MRI) in these patients showed bihemispheric white matter fluid-attenuated inversion recovery (FLAIR) and T2 signal abnormalities, spared U fibers, absent restricted diffusion, absent gadolinium enhancement, increased lactate and decreased N-acetylaspartate peaks on MR spectroscopy [4–8]. Histologically, there was widespread demyelination with vacuolar degeneration and axonal loss [7, 8]. These MRI, nuclear magnetic resonance (NMR) spectroscopy, and histological features resemble heroin-induced toxic leukoencephalopathy (“chasing the dragon”), with the notable exception that the occipital predominance and cerebellar and brainstem involvement typical of heroin leukoencephalopathy was not seen. Both cocaine- and heroin-induced leukoencephalopathies are thought to be associated with mitochondrial dysfunction [6–9].

More recently, a third type of cocaine-induced encephalopathy was proposed by Gilbert who speculated that an adulterant, levamisole, now commonly found in cocaine, may cause acute and recurrent white matter lesions [10]. When used alone as a therapeutic agent, and even after a single dose, levamisole can cause multifocal inflammatory leukoencephalopathy with variable delay in symptom onset ranging from one day to many weeks [11–17]. An immune-mediated mechanism is postulated. However, a recent review of 203 cases of cocaine users who suffered complications attributed to levamisole contamination, such as neutropenia and dermatological disorders in particular, found no cases of leukoencephalopathy [18]. In contrast, two recent cases of cocaine-related leukoencephalopathy were reported wherein the question of levamisole toxicity was specifically raised [19, 20]. In addition, it seems possible that some cases of cocaine-related leukoencephalopathy previously postulated to have a metabolic basis may have been related to levamisole contamination.

The present report describes two cocaine users with multifocal white matter lesions who were treated with steroids and plasma exchange, and subsequently improved. The potential relationship of their presentations to inflammatory white matter disease is discussed.

## Case presentation

### Case 1

**Fig. 1:**
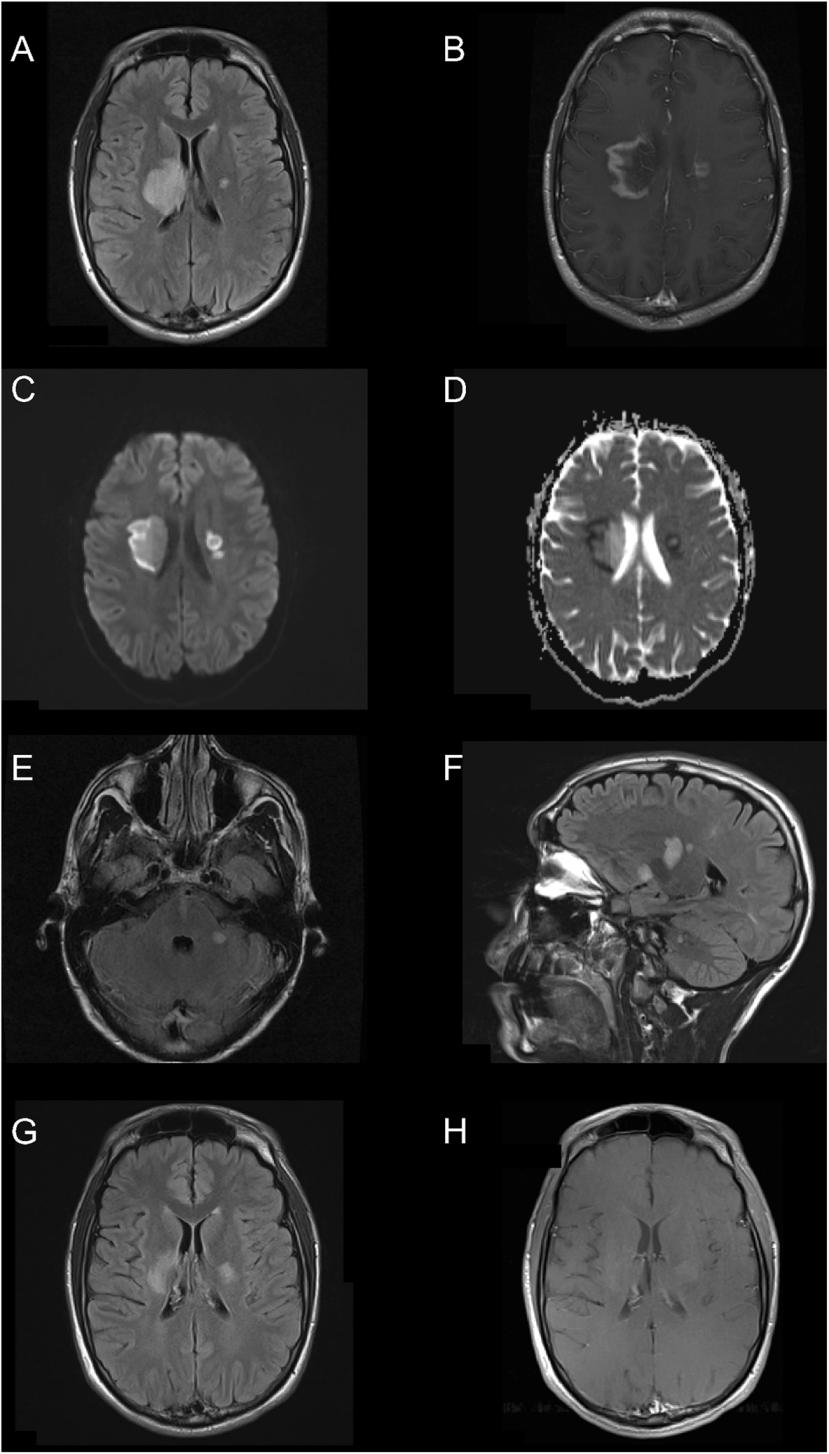
Case 1: MR images of brain lesions. **a** On admission a large area of increased FLAIR signal is present in the right lentiform nucleus, internal capsule, thalamus, corona radiata and centrum semiovale and a small signal focus is present in the left internal capsule/globus pallidus region. **b** A rim of gadolinium enhancement is present. **c** Restricted diffusion is shown by diffusion-weighted imaging in conjunction with the ADC map (**d**). **e** and **f** Five days later FLAIR images show additional lesions in the right medial pons, left cerebellar peduncle, putamen and corona radiata. Five months later, all lesions are smaller (**g**) and non-enhancing (**h**)

A 25-year-old man with history of cocaine use presented to the emergency room with left-sided upper and lower limb weakness as well as sensory impairment involving the face. Symptoms evolved such that he was bed-ridden after 48 h. He had no history of previous neurological or systemic symptoms. Computed tomography (CT) of the brain showed bilateral periventricular hypodensities. CT angiography of cervical and intracranial vessels was unremarkable. An MRI study of the brain demonstrated increased FLAIR signal deep in the right and left hemispheres (Fig. 1a), which also displayed patchy gadolinium enhancement (Fig. 1b) and restricted diffusion (Fig. 1c–d). Five days after admission he developed swallowing difficulty, dysarthria, and confusion. In addition to the previously noted MRI abnormalities, repeat imaging showed new lesions in the left internal and external capsules, putamen and corona radiata, right medial pons and left cerebellar peduncle, with heterogenous gadolinium enhancement (Fig. 1e–f). The neuroradiological impression was that the lesions were most compatible with demyelinating disease. A urine screen was positive for cocaine, cannabinoids, and oxycodone, and negative for amphetamine, methadone, benzodiazepines, and barbiturates. Other investigations included the following normal or negative results: complete blood count, serum electrolytes, urea, creatinine, glucose, erythrocyte sedimentation rate, antinuclear antibodies, rheumatoid factor, perinuclear and cytoplasmic anti-neutrophil cytoplasmic antibodies (pANCA and cANCA respectively), extractable nuclear antigens, lupus inhibitor, C_3_, C_4_, CH_50_, urinalysis, vitamin B_12_, angiotensin converting enzyme level, thyroid stimulating hormone level, free T_3_ and T_4_, cortisol level, lipid profile, very long chain fatty acids, viral serology for HIV, CMV, Hepatitis A, B, and C, Lyme serology, syphilis screen, blood screen for salicylate, acetaminophen, and ethanol. Liver enzymes (AST, ALT and GGT) were mild to moderately elevated and subsequently normalized over a period of six months. Cerebrospinal fluid (CSF) results included: slightly elevated protein at 0.50 g/l (normal 0.20–0.40 g/l), normal glucose and chloride, normal total cell count (3 × 10^6^/l), negative oligoclonal bands, normal IgG index, elevated albumin index at 9.0 (2.7–4.7), negative cryptoccocal antigen, negative cultures for bacteria and fungi, negative polymerase chain reaction (PCR) studies for CMV and HSV 1–2, and normal cytology.

He was treated with intravenous methylprednisolone, 1000 mg daily for five consecutive days, and subsequently underwent plasma exchange. During plasma exchange his neurological condition stabilized and he subsequently achieved good, albeit partial, recovery. At three months follow-up he had mild residual left-sided hemiplegia but was otherwise independent in activities of daily living and starting to return to work. Follow up MRI five months after presentation showed reduction in the size of lesions, no enhancement, and no new lesions (Fig. 1g–h). MRI 21 months after the initial presentation also showed no new lesions (not shown).

### Case 2

**Fig. 2:**
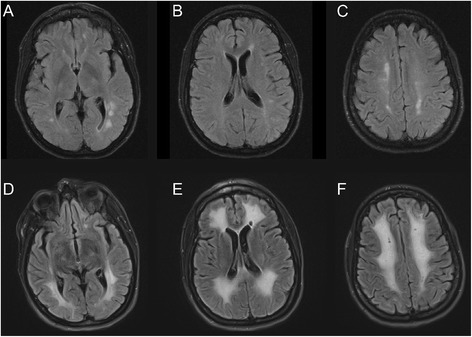
Case 2: MR images (FLAIR) of brain lesions. **a**-**c** On admission foci of increased signal are present in the white matter of the cerebral hemispheres bilaterally, predominantly in a periventricular distribution. **d**-**f** Repeat imaging seven weeks later, after further neurological deterioration, shows prominent interval progression of signal abnormality in the subcortical and periventricular white matter

A 41-year-old woman presented to the emergency room of another hospital with recent confusion and impaired balance. She had been using cocaine daily for seven months. MRI FLAIR images showed multiple white matter signal changes in both cerebral hemispheres in a predominantly periventricular distribution (Fig. 2a–c) sparing the corpus callosum, cerebellum and brainstem. Restricted diffusion was present in many lesions (not shown). Subtle gadolinium enhancement was present in a few lesions (not shown). She was treated with supportive care for one week and then discharged with partial recovery. She resumed cocaine use and was then admitted to our centre six weeks after the previous hospital discharge because of worsening neurological symptoms. She was completely bedridden with inability to walk, incontinence, severely impaired communication skills, behavioural problems, cognitive impairment, and multifocal neurological deficits. MRI FLAIR images showed marked progression compared to the previous study. Confluent white matter abnormalities involved both cerebral hemispheres (Fig. 2d–e). There was subtle scattered restricted diffusion and no gadolinium enhancement. Urine testing was positive for cocaine, oxycodone, opiates, benzodiazepines, tramadol and citalopram, and negative for cannabinoids, amphetamine and methadone. Other investigations included the following normal or negative results: complete blood count, electrolytes, glucose, liver enzymes, thyroid stimulating hormone, T_3_, T_4_, creatinine kinase, angiotensin converting enzyme, HIV, Hepatitis A, B, and C screens, Lyme serology, syphilis screen, blood screen for salicylates, acetaminophen and ethanol. The serum creatinine was elevated (137 micromoles/l) as was the urea (7.6 millimoles/l). Serum albumin was low (29 g/l). The erythrocyte sedimentation rate ranged from 22 to 64 on multiple samplings, C-reactive protein was elevated at 11 mg/l while antinuclear antibodies, pANCA, cANCA, C3, C4, NMDA receptor and anti-thyroid peroxidase antibodies were normal. The vitamin B_12_ level was low at 166 picomole/l (normal >180). Copper, zinc and ceruloplasmin levels were normal. CSF protein was slightly elevated at 0.48 g/l. The glucose, chloride and cell count (4 × 10^6^/l) were normal. No CSF acid fast bacilli were seen and cultures for bacteria and fungi were negative. CSF PCR for CMV and HSV1–2 were negative. She was treated with intravenous methylprednisolone, 1000 mg/day for five days, and plasma exchange. She showed substantial, albeit partial, recovery soon thereafter which continued over the course of several months. At four months follow-up multiple domains of cognitive function were improved but she had some residual deficits in attention, calculation, and visuospatial function. She was independent with respect to walking and most activities of daily living. Follow-up MRI at that time showed improvement of the subcortical and periventricular signal abnormalities and no new lesions. Similarly, no new lesions were seen on MRI 13 months after presentation (not shown).

## Discussion

Only a few cases of cocaine-related leukoencephalopathy have been reported [4–8, 21]. The clinical and MRI features of our two patients suggest that primary vascular mechanisms, which produce the more commonly recognized acute stroke complications of cocaine use, are unlikely. Therefore, the present cases are best considered examples of cocaine-related leukoencephalopathy.

The pattern of white matter involvement in our patients, including both multifocal periventricular and confluent bihemispheric distribution, sparing subcortical U fibers, is similar to imaging results in previous reports of cocaine-induced leukoencephalopathy [4, 6–8]. Because of similarities to heroin-induced leukoencephalopathy, including MR spectroscopy, previous reports speculate that an underlying metabolic derangement, mitochondrial dysfunction in particular, is the substrate of these toxic leukoencephalopathies. Thus, the white matter lesions in our patients may have a similar mechanism.

However, our patients also share features with patients who develop leukoencephalopathy secondary to levamisole [13–17]. Originally developed in the 1960s as an anti-helminthic agent, levamisole also has immune modulating properties and has been used in the treatment immune-mediated and inflammatory disorders such as rheumatoid arthritis, nephritic syndrome, inflammatory bowel disease, and aphthous ulcers, among other conditions. MRI characteristics of levamisole-related brain lesions include hyperintense T2 and FLAIR signal and, variably, diffusion-weighted signal abnormality, gadolinium enhancement and/or surrounding edema [11, 15–17]. The white matter lesions are typically found in subcortical and periventricular white matter, but brainstem and cerebellar involvement can also occur, as was the case in both of our patients. Distinction between levamisole leukoencephalopathy, multiple sclerosis and acute disseminated encephalitis, by imaging features alone can be difficult [16, 17, 21]. Brain biopsy of levamisole-induced lesions demonstrates active demyelination including myelin loss and accumulation of perivascular lymphocytes [12, 16]. Most cases reported in the literature have received steroid [13, 14, 16, 17] or steroid combined with plasmapheresis [11] or immunoglobulin [15], and typically have shown good outcomes. However, if treated late, for instance eight months after levamisole ingestion and onset of symptoms, no improvement is seen [22].

Levamisole has immuno-stimulant properties that increase endogenous opiate levels in the brain and alter monoaminergic function [23]. These properties may be related, at least in part, to its popularity as a cocaine adulterant. It was first detected in cocaine samples in 2003 and by 2009 was present in over 70 % of cocaine specimens tested by the United States Drug Enforcement Agency [24]. In one study, among urine samples testing positive for cocaine, 68 % were also positive for levamisole [24].

Urine levamisole levels were not tested in our patients, and therefore the possibility that levamisole is responsible for their presentation is not proven. It is possible that cocaine alone may have produced white matter lesions through immune-mediated or other mechanisms, independent of levamisole contamination. Cocaine alters endothelial function thereby facilitating migration of immune cells into the central nervous system, increases the secretion of pro-inflammatory cytokines, and enhances the development of acute experimental allergic encephalomyelitis [25].

Similar to patients with levamisole-induced multifocal leukoencephalopathy, steroid treatment of a patient with cocaine-related recurrent leukoencephalopathy was “helpful” according to González-Duarte and Williams [20]. On the other hand recurrent episodes of leukoencephalopathy in another cocaine abuser remitted with and without steroid treatment [6].

## Conclusion

The clinical and MRI improvement observed in our patients, subjected to steroids and plasmapheresis, supports the concept of an immune-mediated mechanism, possibly levamisole-induced, underlying cocaine neurotoxicity in some patients. However, to the best of our knowledge there have been no controlled trials of immunotherapy for the treatment of cocaine-induced leukoencephalopathy. Therefore, extreme caution is warranted interpreting these limited observations, especially given the fact that inflammatory white matter disease (for example multiple sclerosis) can display spontaneous remission. In addition, given the prevalence of cocaine use in the general population, patients presenting with white matter disease suggestive of acute disseminated encephalomyelitis or multiple sclerosis, with or without remissions and relapses, should be carefully reviewed with respect to cocaine use.

## Consent

Both patients provided written informed consent for their cases and accompanying images to be published.

## Abbreviations

MRI: Magnetic resonance imaging
FLAIR: Fluid attenuated inversion recovery
NMR: Nuclear magnetic resonance
CT: Computed tomography
pANCA: Perinuclear anti-neutrophil antibody
cANCA: Cytoplasmic anti-neutrophil cytoplasmic antibody
C3 and C4: Complement component C3 and C4
CH50: Total hemolytic complement
T3: Triiodothyronine
T4: Thyroxine
HIV: Human immunodeficiency virus
CMV: Cytomegalovirus
AST: Aspartate aminotransferase
ALT: Alanine aminotransferase
GGT: Gamma-glutamyltransferase
CSF: Cerebrospinal fluid
HSV: Herpes simplex virus
IgG: Immunoglobulin G
NMDA: N-methyl-D-aspartate
PCR: Polymerase chain reaction
